# Comprehensive ability evaluation and trend analysis of patients with malignant intracranial tumors in the perisurgery period

**DOI:** 10.1002/brb3.2192

**Published:** 2021-09-23

**Authors:** Yaning Wang, Wenlin Chen, Tianrui Yang, Binghao Zhao, Lizhou Zhou, Ziren Kong, Yuekun Wang, Congxin Dai, Yu Wang, Wenbin Ma

**Affiliations:** ^1^ Departments of Neurosurgery Peking Union Medical College Hospital Chinese Academy of Medical Sciences and Peking Union Medical College Beijing China

**Keywords:** brain metastases, Cognitive assessment, glioma, malignant intracranial tumor, quality of life

## Abstract

**Introduction:**

Prognostic situations differ in patients with malignant intracranial tumors. We focused on the quality of life, ability of daily living, and cognitive function of patients in the perisurgery period and investigated the correlation between them and the prognosis of patients.

**Materials and Methods:**

Patients with malignant intracranial tumors admitted to Peking Union Medical College Hospital from May 2018 to August 2020 for surgery were included. The evaluations were performed 6 times in the perisurgery period. The questionnaires for assessment included QLQ‐C30, ADL, and so forth.

**Results:**

A total of 165 patients were included (115 glioma and 50 brain metastases). Patients had their worst performance at the 7‐day postsurgical assessment (EORTC QLQ‐C30, ADL, HAD‐D, Frail Scale, MMSE, MoCA, CSHA‐FI, and NANO) and recovered at the 1‐month postsurgical assessment (*p* < .05). Patients with left‐sided tumors had signiﬁcantly worse cognitive function than patients with right‐sided tumors before surgery and at 7 days, 1 month, and 6 months after surgery (*p* < .05). The scores of QLQ‐C30 and QLQ‐BN20 at 1 month, 3 months, 6 months, and 1 year after surgery were used to reflect the prognosis, and the preoperative MoCA, NANO, CCI, CSHA‐FI, and HAD score might predict the quality of life and nutrition status after operation.

**Conclusion:**

The quality of life and daily living ability of patients with malignant intracranial tumors decreased significantly 7 days after the surgery but recovered 1 month after the surgery. Patients with left hemisphere lesions had a worse cognitive function, while the ADL is associated with short‐term prognosis. The comprehensive evaluation of the perisurgical period can indicate the prognosis of patients and further guide clinical decision‐making.

## Graphical abstract

Our research includes 165 patients with malignant intracranial brain tumor. It mainly focuses on the comprehensive assessments in the perisugery period in order to find the relationship between the results of the assessments and the outcomes of patients. We hope that the conclusion of this research can help clinical workers to treat patients in a more precise and effective way.

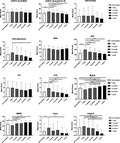

## 1 INTRODUCTION

Malignant intracranial tumors mainly include primary intracranial tumors and brain metastases, of which glioma is the most common primary malignant intracranial tumor in adults. At present, the treatment of glioma patients mainly includes surgery, radiotherapy, chemotherapy, and targeted therapy (Bush et al., 2017; VanderWalde et al., 2016). In addition to primary tumors, the central nervous system is also a common metastatic site of other organ malignant tumors in the whole body. Approximately 20%–40% of patients with tumors in other organs will have brain metastasis, in which lung cancer is the most common primary tumor (Andrevska et al., 2014). Although there is no standard treatment regimen for patients with brain metastases, surgery is the most common therapy for these patients, supplemented by subsequent radiotherapy and drug therapy (Soffietti et al., 2017).

Because the basic performance status of patients with malignant brain tumors is not uniform, the tolerance for surgery, radiotherapy, and drug treatment is different, so the treatment regimen should be selected more specifically for different patients, as different individuals can benefit more from certain treatments. To achieve this goal, determining a universal tool to assess patients’ tolerance to different treatment regimens is a top priority. At present, it has been found that the preoperative status of patients with glioma, including the status of comorbidities, cognitive function, frailty, and so on, is associated with the treatment benefit of the patient, and a positive result can be obtained (Cloney et al., 2016; Ening et al., 2015; Perry et al., 2017). In patients with metastatic tumors, a series of studies on cognitive function before and after radiotherapy have been presented, and the assessment index was used as one of the endpoints of the studies to compare the advantages and disadvantages of different treatment schemes (Chang et al., 2009). The abovementioned studies show that pretreatment assessment, as well as the changing trend of the assessment index in the treatment process, has gradually attracted attention from investigators and clinical workers.

Although there has been an increasing number of studies on pretreatment assessments of patients with brain tumors, there is still a lack of systematic and comprehensive evaluation tools for patients with intracranial malignancies. This project integrated the existing and widely used assessment methods for patients and systematically and comprehensively evaluated all patients with malignant intracranial tumors in this center during the past year. In addition to their clinical information, the evaluation also includes quality of life (QOL), general performance status, emotional state, frailty scale, nutritional status, cognitive function, and comorbidity status. At present, we have obtained the variation trend of the evaluation results, and through systematic follow‐up, we have analyzed the correlation between the evaluation results and the prognosis of patients.

## 2 METHODS

### 2.1 Study design and eligibility criteria

A nonrandomized, prospective, longitudinal study was conducted. The study aimed to evaluate the comprehensive abilities of malignant brain tumor patients, including QOL, the ability of daily living, and cognitive ability. Peking Union Medical College Hospital Review Board approval was obtained before study initiation (registry number JS‐2012), and written informed consent was obtained from each subject. Baseline information was obtained from a medical records review at admission and included demographic information, oncologic history (time of diagnosis, tumor location, nature of the tumor, and treatment history), radiographic data, and other significant medical and surgical histories. The treatment regimen was selected based on the neuro‐oncologists experience. Assessment with scales was performed before brain tumor resection surgery or biopsy surgery, 7 days after surgery, 1 month after surgery, 3 months after surgery, 6 months after surgery, and 1 year after surgery with regular follow‐up. The research staff maintained study contact by interval phone calls at each follow‐up point.

The eligibility criteria were being older than 18 years, having a histologically diagnosed malignant brain tumor, and receiving surgery at Peking Union Medical College Hospital. Patients at the agonal or deep coma stage with cognitive impairment and those who were unconscious during the evaluation process were excluded from this study. Perinatal women were also excluded.

### 2.2 Data collection

Trained researchers conducted the assessment with the scales in person during hospitalization and follow‐ups. The result was recorded in an online database. In addition to the answers to each question, the time usage and general condition of the patients were also recorded. In addition to the questionnaires completed at the follow‐ups, information regarding current systemic treatment and hospitalization was collected. All the data of this research are available.

### 2.3 Assessment questionnaires

The questionnaires used in the assessment, including 11 scales, focused on the cognitive ability, QOL, emotional status, nutritional condition, and general health situation of the patients. The specific tests applied were the Mini‐mental State Examination (MMSE) (Folstein et al., 1975), Montreal Cognitive Assessment (MoCA) (Nasreddine et al., 2005), European Organization for Research and Treatment of Cancer (EORTC) Quality of Life Questionnaire—C30 and BN20 (Aaronson et al., 1993), Hospital Anxiety and Depression Scale (HADS) (Zigmond & Snaith, 1983), Activities of Daily Living (ADL) (Katz et al., 1970), Mini‐Nutritional Assessment (MNA) (Rubenstein et al., 2001), Frail Scale (Abellan van Kan et al., 2008), Charlson Comorbidity Index (CCI) (Ening et al., 2015), Neurologic Assessment in Neuro‐Oncology scale (NANO) (Nayak et al., 2017) and Canadian Study of Health and Aging—Fragility Index (CSHA‐FI) (Cloney et al., 2016). MMSE and MoCA are the most widely used cognitive ability screening scales currently, and the combination of the two tests shows higher accuracy for detecting cognitive dysfunction. A higher score indicates better cognitive status in both MMSE and MOCA, while years of education are associated with the evaluation of cognitive impairment and dementia. For MMSE, the total score is 30 and a score less than 27 suggests cognitive impairment. While for MOCA, the total score is also 30, a score less than 26 suggests cognitive impairment. A score of 18 to 26 indicates mild cognitive impairment, 10–17 indicates moderate cognitive impairment and less than 10 suggests severe cognitive impairment. The EORTC QOL questionnaires are instruments designed for evaluating different aspects defining the QOL of cancer patients, while EORTC QLQ‐C30 is for general tumor patients and EORTC QLQ‐BN20 is for brain tumor patients. A worse quality of life results in higher score, while the last two questions refer to patients’ self‐evaluation in QLQ‐C30 has been evaluated separately in our research, and the score of QLQ‐C30 discussed below refers to the total score minus patients’ self‐evaluation score. ADL can reflect the daily living ability of the patients, and a higher mark suggested worse ability of daily living. MNA can reflect the nutritional level and a lower mark suggested worse state of nutrition. CCI is used for clinical complications assessment, more complications and a higher age result in a higher score. Patient frailty was measured by the Frail Scale and CSHA‐FI, and higher score indicates frailer status in both scales, while the scores fall between 0 and 6 in the Frail Scale and between 0 and 11 in CSHA‐FI. NANO can evaluate the neurologic function and prognosis of glioblastoma multiforme (GBM) patients and has shown significant value in clinical practice, while patients with better neurologic function get a lower grade. In addition, HADS is one of the most commonly used tools for screening anxiety and depression accompanied by medication or physical diseases. Patients who show obvious anxiety or depression tendency tend to get a higher score, while anxiety index and depression index are calculated separately.

Age and education level were recorded to achieve an accurate result. The results of those questionnaires were input into an online database individually, and each of them contained a subset of independent items.

### 2.4 Statistical analysis

Descriptive statistics were conducted to measure the characteristics of the demographic and clinical variables. The Kruskal–Wallis test was conducted to evaluate the relationship between the score of each scale and the assessment time. Spearman's correlation analysis was used to measure the correlation between tumor position and cognitive ability and the correlation between the presurgery results of each scale and the QOL. The results of the scales were individually entered into the analysis as dependent variables. The data were analyzed using SPSS Statistics 24.0, and a two‐sided *p*‐value < .05 was considered statistically significant.

## 3 RESULTS

**FIGURE 1:**
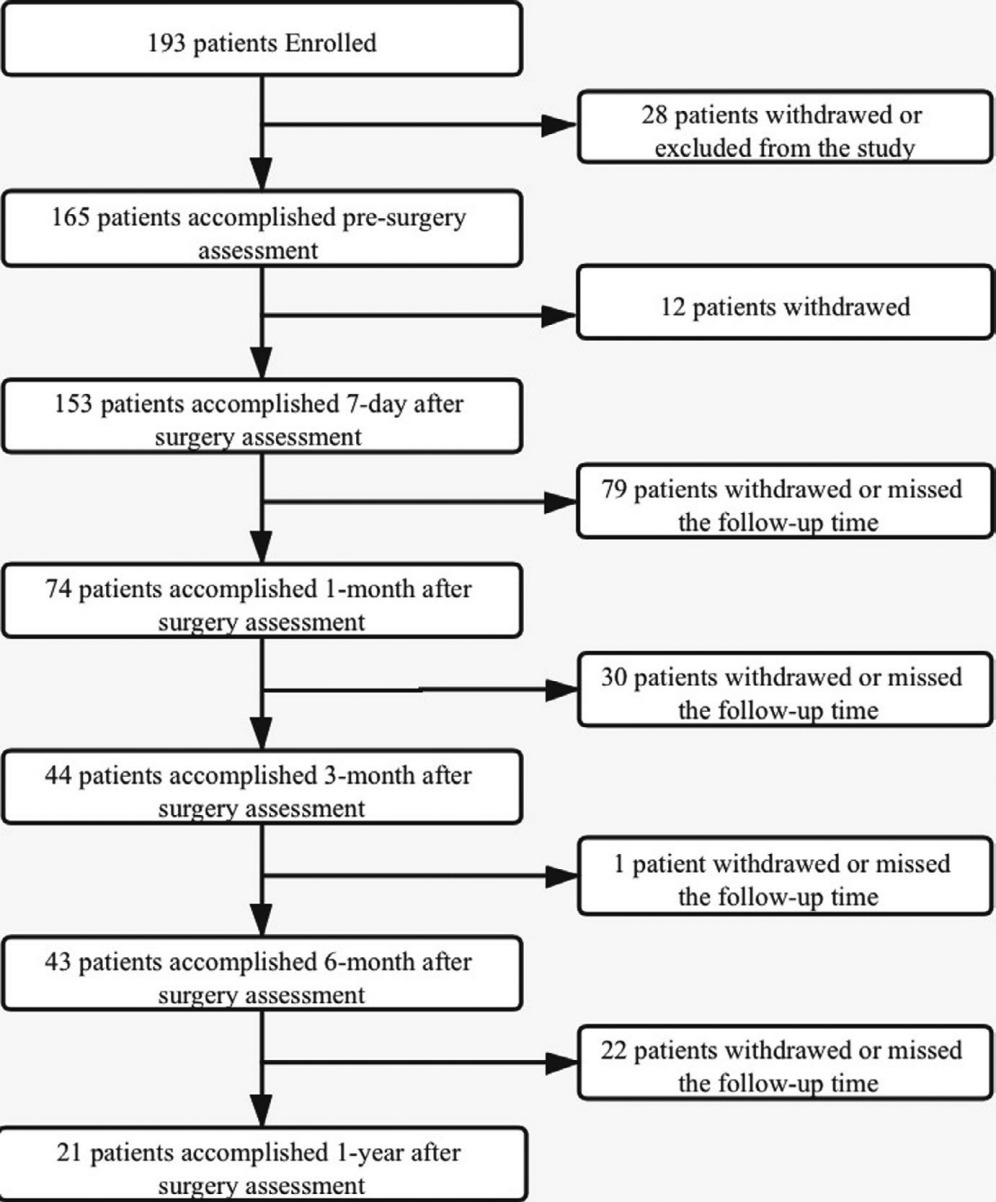
Flow chart of patient enrollment

We enrolled 193 patients with malignant brain tumors in our study until August 2020. One hundred and sixty‐five patients completed the assessment before surgery. Twenty‐eight patients signed the consent form but showed cognitive impairment and were unconscious during the evaluation process or excluded from the study when histopathological findings suggesting nonmalignancy. One hundred and fifty‐three patients completed the assessment 7 days after the surgery. Seventy‐four patients were included in the 1‐month follow‐up, forty‐four patients completed the 3‐month follow‐up, forty‐three patients completed the 6‐month follow‐up, and twenty‐one participants completed all study forms, including the assessment 1 year after surgery (Figure 1).

The demographic information of the patients is summarized in Table 1. Among the 165 patients enroled, there were 89 men and 76 women, with an average age of 52.0 years old (range 19–79). The majority of included patients received 6–12 years of education (*n* = 92, 55.8%). There were 115 glioma patients (39 grade II patients, 30 grade III patients, 31 grade IV patients, and 15 others) and 50 patients with metastatic brain tumors (20 from lung cancer, 8 from breast cancer, 3 from renal cancer, and 19 from other kinds of cancer). Solitary tumors were observed in the majority of patients (*n* = 147, 89%), while multiple lesions appeared in 18 patients. Considering the distribution of the tumor, 64 were located in the left hemisphere, while 96 were located in the right hemisphere of the brain. Meanwhile, 5 patients suffered from bilateral tumors. There were 65 solitary lesions in the frontal lobe, 26 lesions in the temporal lobe, 16 lesions in the parietal lobe, 12 lesions in the occipital lobe, 1 lesion in the insular lobe, and 5 lesions in the infratentorial lobe. The number of tumors locating in more than one lobe was 22. Tumor gross‐total resection (GTR) was performed in 132 patients, subtotal resection (STR) was performed in 11 patients, and neurosurgical biopsy was performed in the remaining 22 patients. Meanwhile, as the participating patients were different in each time period, we put forward the baseline information for every time period in Table 1.

**TABLE 1:** Baseline characteristics of patients of each time period

Demographic characteristics	Presurgery	7 days after surgery	1 month after surgery	3 months after surgery	6 months after surgery	1 year after surgery
Patient number	165	153	74	44	43	21
Sex						
Male	89	81	39	18	20	9
Female	76	72	35	26	23	12
Age						
18–30	11	10	7	5	7	3
31–50	60	58	29	18	20	12
51–65	74	67	32	18	11	3
66 and older	20	18	6	3	5	3
Years of education						
<6	25	22	10	7	4	2
6–12	92	87	49	27	30	14
>12	48	44	15	10	9	5
Tumor type						
Glioma	115	106	51	31	33	17
Metastatic tumor	50	47	23	13	10	4
Number of tumor						
Solitary	147	136	68	40	40	19
Multiple	18	17	6	4	3	2
Side of the tumor						
Left hemisphere	64	60	33	22	18	9
Right hemisphere	96	89	40	22	25	12
Bilateral	5	4	1	0	0	0
Tumor location (Solitary Tumor)						
Frontal lobe	65	62	34	23	20	12
Temporal lobe	26	24	9	6	5	2
Parietal lobe	16	16	8	3	7	1
Occipital lobe	12	11	5	3	3	2
Insular lobe	1	1	0	0	0	0
Frontoparietal lobes	3	3	3	1	2	0
Frontotemporal lobe	7	6	3	2	2	1
Parietooccipital lobe	6	3	3	1	1	1
Occipitotemporal lobe	5	5	1	0	0	0
Parietotemporal lobe	1	1	0	0	0	0
Infratentorial	5	4	2	1	0	0
Surgery option						
gross‐total resection	132	124	62	37	35	16
subtotal resection	11	11	6	2	5	3
biopsy	22	18	6	5	3	2

**FIGURE 2:**
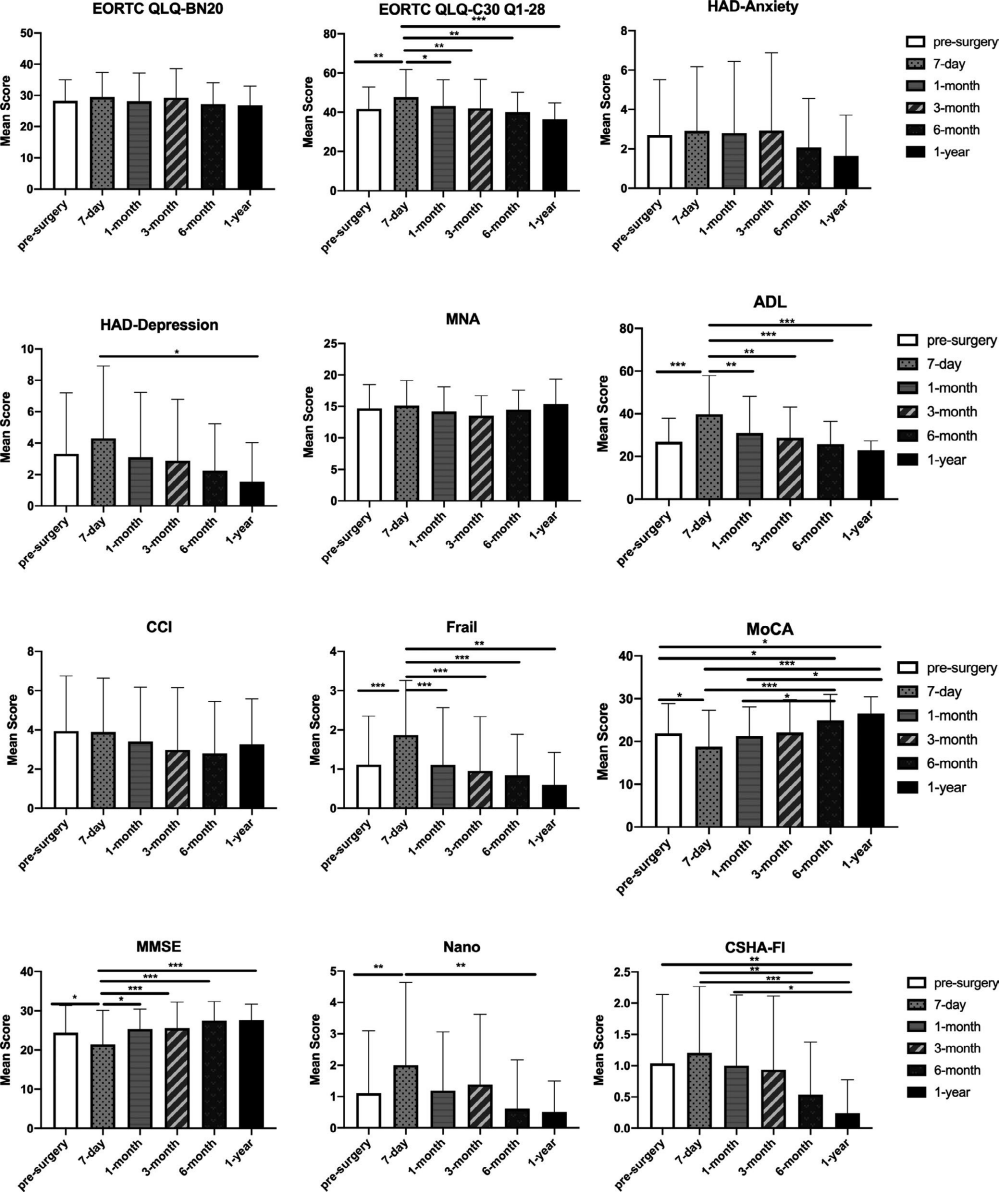
Mean scores of each assessment scale before surgery and follow‐up assessment time period, Significant difference is marked by *, *, *p* < .05; **, *p* < .01; ***, *p* < .001

The correlation between the time period and the result of the assessment was obvious. Due to the nonnormal nature of the data, Kruskal–Wallis analysis was conducted. The results of EORTC QLQ‐C30, ADL, HAD‐D, Frail Scale, MMSE, MoCA, CSHA‐FI, and NANO showed the worst situation of the patients during the 7‐day postsurgical assessment (with *p* < .05) (Figure 2). A slight declining trend of ability was also observed in EORTC QLQ‐BN20 7 days after surgery, while HADS‐A, CCI, and MNA did not show significant variation. In the 1‐month postsurgical assessment, all abilities of the patient basically returned to the preoperative level (*p* < .05). The results were rather stable 3 months postsurgery. Interestingly, there were significant differences in the 1‐year postsurgical assessment of MoCA and CSHA‐FI with the result of other time periods of the corresponding scale. For the cognitive ability measurement, we concluded the result of MoCA and MMSE in each stage of brain malignant tumor patient assessment (Table S1). A parallel phenomenon is observed in MoCA and MMSE, the rate of cognitively normal patients reached the bottom at 7 days postsurgery assessment and showed a steady escalation in the following assessments. This phenomenon might be caused by the small proportion of patients who accomplished this evaluation, and the patients able to accomplish the scale evaluation 1 year after surgery usually showed a better recognition performance and general health situation.

**FIGURE 3:**
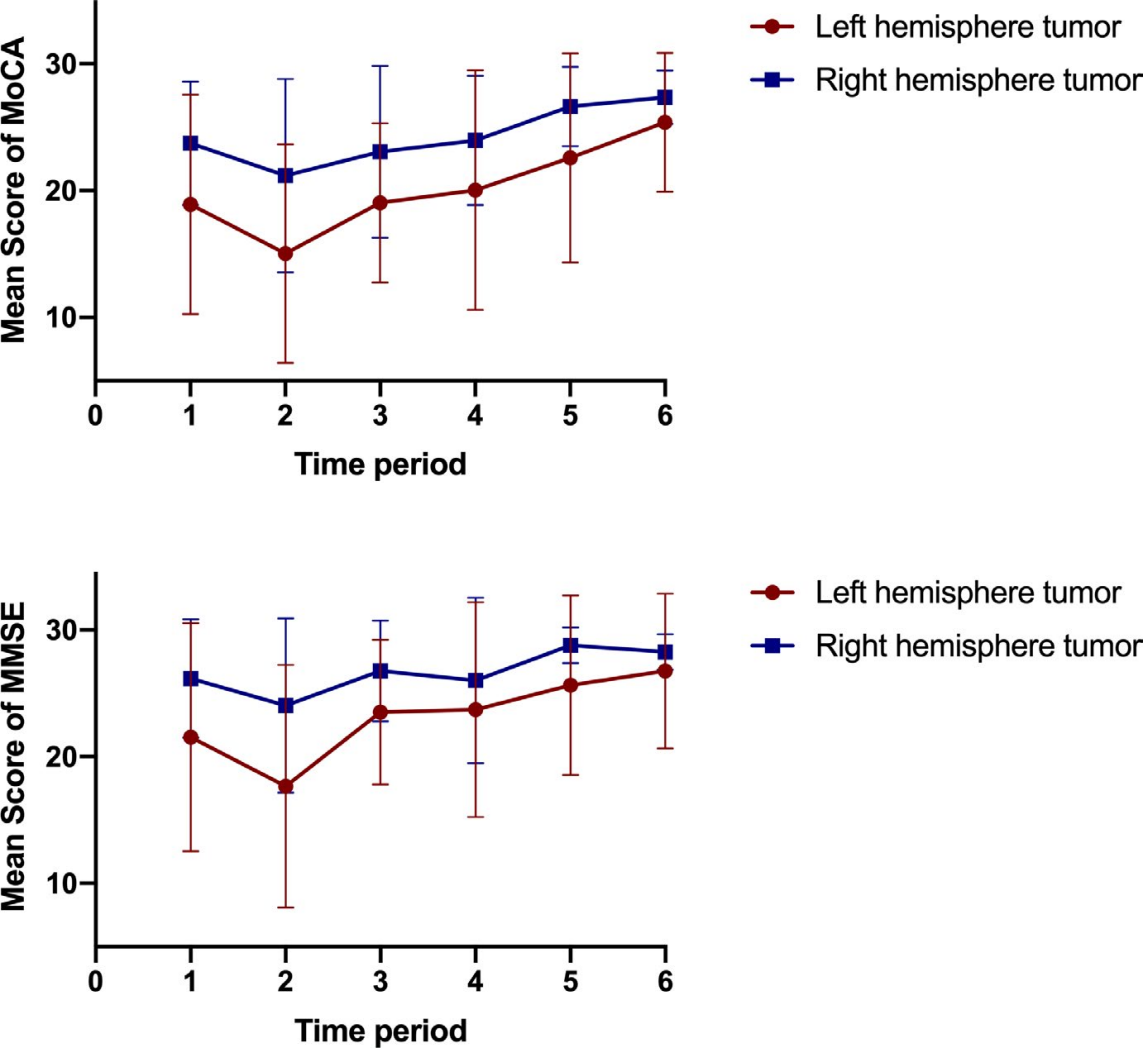
Differences in the cognitive abilities of patients with tumors located in the left and right hemispheres. Cognitive ability was assessed by MoCA (a) and MMSE (b) (*p* < .05)

Tumor location was significantly correlated with the cognitive ability of patients. Compared with patients with neoplasms in the right hemisphere of the brain, patients with malignant tumors in the left hemisphere showed remarkably weaker cognitive ability (measured by MoCA and MMSE) before surgery, 7 days after surgery, 1 month after surgery, and 6 months after surgery, while the result in 1‐year after surgery assessment did not show statistical significance (*p* < .05) (Figure 3).

In this study, we regarded the EORTC QLQ‐C30 and QLQ‐BN20 scales at 1 month, 3 months, 6 months, and 1 year after surgery as prognostic criteria, reflecting the perioperative prognosis of patients. The scores of MNA at the same time period after surgery are analyzed as prognostic criteria measuring the patients’ perioperative nutritional status. We analyzed the relevance between the assessment results of each scale in the preoperative period and the perioperative prognosis situation (Tables [Link], [Link]). Due to the discrete and nonnormal nature of the data, Spearman's correlation analysis was conducted. Among the assessment scales, perioperative CSHA‐FI and NANO were correlated with the QLQ‐BN20 3‐month postsurgical scores (*p* < .05), while MoCA was correlated with the QLQ‐BN20 1‐year postsurgical score. As for the QLQ‐C30 scale, the perioperative score of MoCA was related to 1‐month and 3‐month postsurgical scores, the perioperative score of CCI were correlated with the 3‐month after surgery score and the perioperative result of MNA was associated with the 1‐year result of QLQ‐C30. Meanwhile, the perioperative score of HAD‐A might predict the 1‐month postsurgical score of MNA, and HAD‐D and MoCA were related to the result of MNA 6 months after surgery.

To avoid the influence of the varied number of patients at each time period, we analyzed the patients who received the assessment 3 months after surgery and 6 months after surgery, respectively, to explore the value of the scale assessment in predicting the prognosis in the same population. In the 3‐month postoperation group, perioperative results of ADL and Frail scale were discovered to be correlated with 1‐month postsurgical score of QLQ‐BN20 and the 3‐month postsurgical result of QLQ‐C30, and perioperative QLQ‐C30 score might reflect 6‐month postoperation result of MNA. Perioperative results of CCI was correlated with 3‐month postsurgical outcome of QLQ‐C30, and the score CSHA‐FI and NANO might predict the result of QLQ‐C30 1‐year after surgery (Tables [Link], [Link]). In the 6‐month postoperation group, perioperative result of ADL was also discovered as a possible predictor for 3‐month and 6‐month postsurgical outcome of QLQ‐C30, while MoCA and MMSE suggested the result of QLQ‐C30 and QLQ‐BN20 1‐month after surgery. Also, perioperative result of MoCA and MNA were correlated with the 6‐month postoperation result of QLQ‐BN20. The perioperative outcomes of QLQ‐BN20, HAD‐A, HAD‐D, and CSHA were significantly related to the 6‐month after surgery assessment result of MNA, MMSE also indicated the result of MNA 3 months postoperation (Tables [Link], [Link]).

## 4 DISCUSSION

In this study, all included patients were evaluated before the operation, and at 7 days, 1 month, 3 months, 6 months, and 1 year after the operation. The evaluation data showed that the variation in all the evaluation items was related to the time changes, which is the first conclusion of this study. This result was consistent with those of previous multicenter randomized controlled studies. Paul D. Brown et al. evaluated the cognitive function of 213 patients with brain metastases receiving radiotherapy in 34 centers, as well as the QOL and ADL of some patients (Brown et al., 2016). The evaluation nodes were before operation and 6 weeks, 3 months, 6 months, 9 months, 12 months, 16 months, 2 years, 3 years, 4 years, and 5 years after operation. In this research, cognitive function was less affected using stereotactic radiotherapy alone. Taking cognitive function as one of the endpoints of the study and evaluating the effectiveness of different treatment regimens can be the next step of our study.

In the past, Vyshak Alva Venur et al. summarized the studies on the construction of prognosis prediction scoring systems in patients with brain metastases (Venur & Ahluwalia, 2015). Among all the scoring systems, the Recursive Partitioning Analysis system (RPA system) (Gaspar et al., 1997), Rotterdam Score system (Lagerwaard et al., 1999), Graded Prognostic Assessment system (GPA system) (Sperduto et al., 2008), and disease‐specific Graded Prognostic Assessment system (ds‐GPA system) (Sperduto et al., 2010) are some of the most convincing, as they included more patients than others. These systems include the performance status, the number of intracranial lesions, age, the condition of primary tumors, and the condition of other metastases in the whole body as predictors of the survival time of patients with brain tumors. In addition to the items mentioned above, our study also included the QOL, ADL, mental condition, frailty scale, nutritional status, cognitive function, and comorbidity status, which makes the evaluation a more comprehensive, multidimensional evaluation system. As a prospective study, all patients were followed up periodically; therefore, after obtaining more patients' survival data, establishing a prognosis prediction scoring system is also the direction of future efforts of our program.

There were 115 patients with glioma and 31 patients with brain metastases included in our study. However, considering that the number of cases of each pathological type and grade is still small, the significance of statistical analysis is unclear, so no comparative analysis was performed for patients with different pathological types or grades. However, in previous studies, some researchers have compared the cognitive status of patients with different pathological types of intracranial mass lesions, including gliomas, meningiomas, brain metastases, and lymphomas (Hoffermann et al., 2017; Kayl & Meyers, 2003). However, the results showed that the pathological types of intracranial tumors had no significant effect on the cognitive function of patients.

The second conclusion of this study suggests that the locations of lesions are significantly related to cognitive function, which has also been verified in previous studies. In 2015, Kyle R. Noll et al. (Noll et al., 2015) studied whether the location of lesions (i.e., on the left or right side) was associated with the cognitive function of patients. In this study, 45 patients with lesions in the left temporal lobe and 19 patients with lesions in the right temporal lobe were included. The results showed that the location of the lesion did have an impact on the cognitive function of the patients. The damage to naming ability in the cognitive function of patients with lesions in the left temporal lobe was significantly higher than that in patients with lesions in the right temporal lobe. This research team further expanded the sample size in 2016 and analyzed the influence of the location of lesions on cognition again (Noll et al., 2016). The results showed that the cognitive dysfunction of patients with lesions in the right temporal lobe was less than that in the left side, but there was obvious impairment of verbal memory in this group of patients.

In addition to the correlation analysis between cognitive function and the location of the lesion, the cognitive function of the patients was also associated with survival time. Perry JR et al. (Perry et al., 2017) found that elderly patients with high MMSE scores could obtain longer overall survival (OS). Johnson DR et al. (Johnson et al., 2012) suggested that language function, executive function, and attention can be used as independent predictors of prognosis in elderly patients with GBM. In view of the long survival time of patients with low‐grade gliomas, a considerable number of studies carried out a long‐term follow‐up evaluation of the cognitive function of these patients. Linda Douw et al. (Douw et al., 2009) evaluated the cognitive function of 195 patients with low‐grade gliomas for up to 12 years and regarded cognitive function as one of the endpoints of the study, indicating the importance of cognitive function. The above conclusions fully explain the necessity of continuous cognitive function assessment in patients with malignant intracranial tumors.

In recent years, QOL has become an important outcome index in evaluating a treatment plan gradually. The relationship between the QOL and survival time of patients with glioma has already been explored. Although QOL cannot be used as an independent prognostic factor for the survival time of patients with brain tumors, patients with low scores have a higher risk of language disorder, confusion of thinking, and limited motor function after operation (Peters et al., 2014). Our study used the QOL scores of patients after operation with malignant intracranial tumors as outcome indexes, and other items evaluated before operation were analyzed. The results showed that the ability of daily living before operation was significantly correlated with the QOL score (*p* < .05), suggesting that it is very necessary for us to pay attention to the ability of daily living before operation, which is the third conclusion of this study. This conclusion is also consistent with those of previous studies. Vander Walde NA. et al. (VanderWalde et al., 2017) carried out a comprehensive assessment of elderly cancer patients, including the ability of daily living. The results showed that the index was significantly related to the QOL of these patients, but whether this index can be used as an independent risk factor to predict the survival time needs to be further studied.

In addition to the three conclusions mentioned above, our study also evaluated the mental and emotional status, frailty scale, nutritional status, and comorbidity status of the patients before operation，the correlation between these indexes and QOL was further analyzed, confirming the prognostic value of these indexes for intracranial malignant tumors. These indicators have also been studied in previous studies. It can be seen that they are all related to the prognosis of patients with malignant intracranial tumors, especially in geriatric patients (Noll et al., 2017). Future studies can stratify age and carry out related subgroup studies on the basis of the further expansion of the sample size.

There are also some limitations of this research. Firstly, the number of cases of each pathological type and grade is small, so researchers cannot complete the statistical analysis between them, which needs to further expand the sample size. Also, only a part of patients has completed all the evaluation within a year, so it will be an effort to further improve the follow‐up mechanism and obtain more comprehensive data in the future. Secondly, the overall survival (OS) data are being collected, so this article doesn't analyze the association between the results of the evaluation and the OS. Although OS is an irreplaceable item, there is an increasing number of studies that use the cognitive function and QOL to be the outcome indicators, so does this study. However, researchers will continue to follow the enrolled patients and obtain their survival data, further confirming the value of preoperative evaluation based on this research.

## 5 CONCLUSION

Our research demonstrated that all the evaluation items were significantly related to time changes. The scores of several items (QLQ‐C30, ADL, HAD‐D, and Frail Scale) showed the worst situation in the 7‐day postsurgical assessment. And they will return to the preoperative level and become stable gradually according to the 1‐month, 3‐month, 6‐month, and 1‐year postsurgical assessments. The data also suggest that the locations of lesions are significantly related to cognitive function, which is becoming an important outcome indicator of patients with intracranial malignant tumors. Finally, this research showed that the ADL score before operation was correlated to the QOL score (*p *＜ .05). It emphasizes the importance of preoperative ADL evaluation and confirms the significance of preoperative comprehensive ability evaluations.

## CONFLICT OF INTEREST

All authors have no conflicts of interest to disclose.

## AUTHOR CONTRIBUTIONS

All authors designed and conducted this research. Yaning Wang and Wenlin Chen wrote the paper. Wenbin Ma and Yu Wang had primary responsibility for the final content. All authors read and approved the final manuscript. Notably, Yaning Wang and Wenlin Chen equally share the first authorship; Wenbin Ma and Yu Wang equally share the corresponding authorship.

### PEER REVIEW

The peer review history for this article is available at https://publons.com/publon/10.1002/brb3.2192.

## Supporting information

Table S1

Table S2

Table S3

Table S4

Table S5

Table S6

Table S7

Table S8

Table S9

Table S10

## Data Availability

The data of this research are available on request of the corresponding authors.
