# Supplementary material for: Comprehensive ability evaluation and trend analysis of patients with malignant intracranial tumors in the perisurgery period
Source: Brain Behav. 2021 Sep 23;11(11):e02192. doi: 10.1002/brb3.2192 (PMC8613416; doi:10.1002/brb3.2192)
Supplement: Supplementary file 1 — Table S1 [file BRB3-11-e02192-s005.docx]

Table S1 Result of cognitive ability in each stage of brain malignant tumor patient assessment. Cognitive ability was measured by MoCA and MMSE.

|  |  | Before surgery | 7 days after surgery | 1 month after surgery | 3 months after surgery | 6 months after surgery | 1 year after surgery |
| --- | --- | --- | --- | --- | --- | --- | --- |
| MoCA |  |  |  |  |  |  |  |
|  | Normal (≥26) | 59 (37.8%) | 36 (24.7%) | 27 (36.5%) | 19 (42.2%) | 26 (63.4%) | 16 (84.2%) |
|  | Mild cognitive impairment (18-25) | 61(39.1%) | 51(34.9%) | 23 (31.1%) | 17 (37.8%) | 11 (26.8%) | 2 (10.5%) |
|  | Moderate cognitive impairment (10-17) | 27 (17.3%) | 34 (23.3%) | 19 (25.7%) | 6 (13.3%) | 1 (2.4%) | 1 (5.3%) |
|  | Severe cognitive impairment (<10) | 9 (5.8%) | 25 (17.1%) | 5 (6.7%) | 3 6.7(%) | 3 (7.3%) | 0 |
| MMSE |  |  |  |  |  |  |  |
|  | Normal (≥27) | 85 (54.1%) | 51 (35.2%) | 39 (52.7%) | 31 (68.9%) | 34 (82.9%) | 17 (85.0%) |
|  | Mild cognitive impairment (21-26) | 45 (28.7%) | 46 (31.7%) | 22 (29.7%) | 6 (13.3%) | 4 (9.8%) | 2 (10.0%) |
|  | Moderate cognitive impairment (10-20) | 16 (10.2%) | 29 (20.0%) | 13 (17.6%) | 6 (13.3%) | 2 (4.9%) | 1 (5.0%) |
|  | Severe cognitive impairment (<10) | 11 (7.0%) | 19 (13.1%) | 0 | 2 (4.5%) | 1 (2.4%) | 0 |
