# Supplementary material for: Comprehensive ability evaluation and trend analysis of patients with malignant intracranial tumors in the perisurgery period
Source: Brain Behav. 2021 Sep 23;11(11):e02192. doi: 10.1002/brb3.2192 (PMC8613416; doi:10.1002/brb3.2192)
Supplement: Supplementary file 2 — Table S2 [file BRB3-11-e02192-s001.docx]

| QLQ-BN20 Correlation analysis | | | | | | | | |
| --- | --- | --- | --- | --- | --- | --- | --- | --- |
|  | 1-month after surgery | | 3-month after surgery | | 6-month after surgery | | 1-year after surgery | |
|  | Correlation coefficient | Significance | Correlation coefficient | Significance | Correlation coefficient | Significance | Correlation coefficient | Significance |
| ADL | 0.023 | 0.843 | 0.237 | 0.109 | -0.090 | 0.568 | 0.035 | 0.891 |
| HAD-A | -0.084 | 0.469 | -0.208 | 0.161 | 0.019 | 0.902 | -0.079 | 0.756 |
| HAD-D | -0.087 | 0.452 | 0.213 | 0.150 | 0.120 | 0.442 | 0.084 | 0.741 |
| Frail | -0.011 | 0.928 | -0.024 | 0.873 | 0.072 | 0.648 | 0.285 | 0.253 |
| MNA | -0.010 | 0.929 | -0.087 | 0.562 | 0.215 | 0.166 | 0.393 | 0.107 |
| MoCA | -0.076 | 0.509 | -0.017 | 0.907 | 0.175 | 0.262 | **0.540** | **0.021** |
| MMSE | -0.070 | 0.547 | -0.172 | 0.249 | 0.271 | 0.078 | -0.041 | 0.871 |
| CCI | -0.023 | 0.840 | 0.242 | 0.101 | -0.180 | 0.248 | -0.342 | 0.164 |
| CSHA | 0.206 | 0.072 | **0.312** | **0.033** | 0.047 | 0.764 | -0.047 | 0.853 |
| NANO | 0.011 | 0.925 | **-0.332** | **0.023** | 0.043 | 0.787 | 0.164 | 0.515 |

Table S2 Correlation of pre-surgery evaluation score and perioperative prognosis situation. Prognosis was measured by QLQ-BN20 in 1-month, 3-month, 6-month and 1-year after surgery(p<0.05).
