# Supplementary material for: Comprehensive ability evaluation and trend analysis of patients with malignant intracranial tumors in the perisurgery period
Source: Brain Behav. 2021 Sep 23;11(11):e02192. doi: 10.1002/brb3.2192 (PMC8613416; doi:10.1002/brb3.2192)
Supplement: Supplementary file 3 — Table S3 [file BRB3-11-e02192-s004.docx]

| QLQ-C30 Correlation analysis | | | | | | | | |
| --- | --- | --- | --- | --- | --- | --- | --- | --- |
|  | 1-month after surgery | | 3-month after surgery | | 6-month after surgery | | 1-year after surgery | |
|  | Correlation coefficient | Significance | Correlation coefficient | Significance | Correlation coefficient | Significance | Correlation coefficient | Significance |
| ADL | 0.146 | 0.218 | -0.104 | 0.498 | -0.128 | 0.408 | 0.088 | 0.711 |
| HAD-A | -0.136 | 0.253 | -0.117 | 0.446 | -0.122 | 0.432 | -0.333 | 0.152 |
| HAD-D | 0.070 | 0.556 | 0.045 | 0.768 | 0.248 | 0.104 | 0.047 | 0.843 |
| Frail | 0.114 | 0.338 | 0.118 | 0.440 | 0.089 | 0.566 | 0.191 | 0.420 |
| MNA | 0.140 | 0.239 | -0.190 | 0.211 | 0.005 | 0.975 | **0.534** | **0.015** |
| MoCA | **-0.260** | **0.026** | **0.301** | **0.045** | 0.063 | 0.683 | -0.305 | 0.192 |
| MMSE | -0.092 | 0.438 | 0.212 | 0.163 | 0.014 | 0.928 | -0.191 | 0.420 |
| CCI | -0.179 | 0.130 | -0.131 | 0.390 | **-0.433** | **0.003** | -0.072 | 0.762 |
| CSHA | -0.128 | 0.279 | 0.080 | 0.603 | -0.030 | 0.848 | -0.233 | 0.322 |
| NANO | 0.006 | 0.960 | 0.095 | 0.536 | 0.249 | 0.102 | 0.260 | 0.269 |

Table S3 Correlation of pre-surgery evaluation score and perioperative prognosis situation. Prognosis was measured by QLQ-C30 in 1-month, 3-month, 6-month and 1-year after surgery(p<0.05).
