# Supplementary material for: Comprehensive ability evaluation and trend analysis of patients with malignant intracranial tumors in the perisurgery period
Source: Brain Behav. 2021 Sep 23;11(11):e02192. doi: 10.1002/brb3.2192 (PMC8613416; doi:10.1002/brb3.2192)
Supplement: Supplementary file 4 — Table S4 [file BRB3-11-e02192-s003.docx]

| MNA Correlation analysis | | | | | | | | |
| --- | --- | --- | --- | --- | --- | --- | --- | --- |
|  | 1-month after surgery | | 3-month after surgery | | 6-month after surgery | | 1-year after surgery | |
|  | Correlation coefficient | Significance | Correlation coefficient | Significance | Correlation coefficient | Significance | Correlation coefficient | Significance |
| QLQ BN20 | -0.159 | 0.176 | 0.139 | 0.351 | -0.051 | 0.746 | 0.154 | 0.518 |
| QLQ C30 | -0.210 | 0.073 | -0.012 | 0.935 | -0.164 | 0.293 | 0.180 | 0.449 |
| ADL | -0.054 | 0.650 | 0.014 | 0.927 | 0.122 | 0.437 | -0.043 | 0.858 |
| HAD-A | **-0.263** | **0.024** | -0.062 | 0.679 | -0.010 | 0.950 | -0.281 | 0.231 |
| HAD-D | -0.101 | 0.393 | 0.067 | 0.654 | **-0.361** | **0.017** | 0.347 | 0.134 |
| Frail | 0.160 | 0.174 | 0.012 | 0.935 | -0.145 | 0.352 | -0.042 | 0.862 |
| MoCA | -0.042 | 0.722 | 0.175 | 0.240 | **0.303** | **0.048** | -0.251 | 0.286 |
| MMSE | 0.026 | 0.826 | -0.050 | 0.738 | 0.256 | 0.097 | 0.011 | 0.962 |
| CCI | -0.114 | 0.334 | -0.157 | 0.292 | 0.054 | 0.731 | -0.220 | 0.352 |
| CSHA | -0.008 | 0.944 | -0.087 | 0.561 | -0.079 | 0.613 | 0.245 | 0.297 |
| NANO | 0.158 | 0.179 | 0.002 | 0.991 | -0.043 | 0.785 | 0.351 | 0.129 |

Table S4 Correlation of pre-surgery evaluation score and perioperative nutritional status situation. Nutritional status was measured by MNA in 1-month, 3-month 6-month and 1-year after surgery(p<0.05).
