# Supplementary material for: Comprehensive ability evaluation and trend analysis of patients with malignant intracranial tumors in the perisurgery period
Source: Brain Behav. 2021 Sep 23;11(11):e02192. doi: 10.1002/brb3.2192 (PMC8613416; doi:10.1002/brb3.2192)
Supplement: Supplementary file 5 — Table S5 [file BRB3-11-e02192-s009.docx]

| QLQ-BN20 Correlation analysis | | | | | | | | |
| --- | --- | --- | --- | --- | --- | --- | --- | --- |
|  | 1-month after surgery | | 3-month after surgery | | 6-month after surgery | | 1-year after surgery | |
|  | Correlation coefficient | Significance | Correlation coefficient | Significance | Correlation coefficient | Significance | Correlation coefficient | Significance |
| ADL | 0.077 | 0.660 | **0.417** | **0.005** | 0.226 | 0.312 | 0.033 | 0.911 |
| HAD-A | -0.082 | 0.641 | -0.066 | 0.673 | 0.035 | 0.876 | 0.153 | 0.602 |
| HAD-D | -0.204 | 0.239 | -0.180 | 0.249 | 0.258 | 0.246 | 0.044 | 0.881 |
| Frail | 0.186 | 0.285 | **0.562** | **0.000** | -0.109 | 0.628 | 0.235 | 0.418 |
| MNA | 0.047 | 0.790 | 0.187 | 0.229 | -0.039 | 0.863 | 0.101 | 0.732 |
| MoCA | -0.020 | 0.870 | 0.074 | 0.636 | -0.105 | 0.643 | 0.062 | 0.833 |
| MMSE | 0.069 | 0.692 | 0.108 | 0.491 | -0.054 | 0.813 | -0.109 | 0.711 |
| CCI | -0.038 | 0.828 | 0.248 | 0.128 | 0.205 | 0.361 | 0.327 | 0.254 |
| CSHA | 0.094 | 0.592 | 0.082 | 0.610 | -0.097 | 0.667 | 0.368 | 0.195 |
| NANO | -0.018 | 0.916 | 0.254 | 0.105 | 0.140 | 0.535 | 0.265 | 0.361 |

Table S5 Correlation of pre-surgery evaluation score and perioperative prognosis situation of patients finished the 3-month after surgery assessment. Prognosis was measured by QLQ-BN20 in 1-month, 3-month, 6-month and 1-year after surgery(p<0.05).
