# Supplementary material for: Comprehensive ability evaluation and trend analysis of patients with malignant intracranial tumors in the perisurgery period
Source: Brain Behav. 2021 Sep 23;11(11):e02192. doi: 10.1002/brb3.2192 (PMC8613416; doi:10.1002/brb3.2192)
Supplement: Supplementary file 6 — Table S6 [file BRB3-11-e02192-s007.docx]

| QLQ-C30 Correlation analysis | | | | | | | | |
| --- | --- | --- | --- | --- | --- | --- | --- | --- |
|  | 1-month after surgery | | 3-month after surgery | | 6-month after surgery | | 1-year after surgery | |
|  | Correlation coefficient | Significance | Correlation coefficient | Significance | Correlation coefficient | Significance | Correlation coefficient | Significance |
| ADL | -0.010 | 0.954 | **0.443** | **0.003** | 0.091 | 0.686 | 0.019 | 0.945 |
| HAD-A | 0.050 | 0.770 | -0.108 | 0.491 | 0.016 | 0.944 | -0.126 | 0.654 |
| HAD-D | -0.054 | 0.755 | -0.138 | 0.379 | 0.140 | 0.534 | -0.162 | 0.564 |
| Frail | 0.205 | 0.231 | **0.519** | **0.000** | -0.248 | 0.265 | 0.358 | 0.190 |
| MNA | 0.025 | 0.886 | 0.143 | 0.360 | 0.010 | 0.966 | 0.024 | 0.932 |
| MoCA | 0.048 | 0.782 | 0.007 | 0.967 | 0.166 | 0.460 | 0.038 | 0.894 |
| MMSE | 0.280 | 0.098 | 0.061 | 0.696 | 0.182 | 0.419 | -0.011 | 0.969 |
| CCI | -0.141 | 0.412 | **0.406** | **0.010** | -0.025 | 0.913 | 0.269 | 0.332 |
| CSHA | 0.148 | 0.390 | 0.019 | 0.908 | -0.313 | 0.156 | **0.569** | **0.027** |
| NANO | 0.027 | 0.876 | 0.088 | 0.581 | -0.030 | 0.895 | **0.597** | **0.019** |

Table S6 Correlation of pre-surgery evaluation score and perioperative prognosis situation of patients finished the 3-month after surgery assessment. Prognosis was measured by QLQ-C30 in 1-month, 3-month, 6-month and 1-year after surgery(p<0.05).
