# Supplementary material for: Comprehensive ability evaluation and trend analysis of patients with malignant intracranial tumors in the perisurgery period
Source: Brain Behav. 2021 Sep 23;11(11):e02192. doi: 10.1002/brb3.2192 (PMC8613416; doi:10.1002/brb3.2192)
Supplement: Supplementary file 7 — Table S7 [file BRB3-11-e02192-s006.docx]

| MNA Correlation analysis | | | | | | | | |
| --- | --- | --- | --- | --- | --- | --- | --- | --- |
|  | 1-month after surgery | | 3-month after surgery | | 6-month after surgery | | 1-year after surgery | |
|  | Correlation coefficient | Significance | Correlation coefficient | Significance | Correlation coefficient | Significance | Correlation coefficient | Significance |
| QLQ BN20 | 0.040 | 0.821 | -0.298 | 0.055 | 0.217 | 0.332 | 0.122 | 0.677 |
| QLQ C30 | -0.068 | 0.696 | -0.244 | 0.110 | **0.475** | **0.025** | -0.265 | 0.361 |
| ADL | -0.029 | 0.870 | -0.256 | 0.094 | 0.074 | 0.744 | -0.039 | 0.896 |
| HAD-A | -0.011 | 0.948 | -0.046 | 0.769 | 0.060 | 0.791 | 0.332 | 0.246 |
| HAD-D | -0.108 | 0.535 | -0.025 | 0.876 | -0.012 | 0.957 | 0.366 | 0.198 |
| Frail | -0.300 | 0.080 | -0.164 | 0.286 | 0.402 | 0.064 | -0.400 | 0.156 |
| MoCA | -0.095 | 0.589 | -0.040 | 0.797 | 0.127 | 0.573 | 0.169 | 0.563 |
| MMSE | 0.092 | 0.598 | 0.035 | 0.826 | -0.140 | 0.535 | -0.091 | 0.757 |
| CCI | 0.078 | 0.657 | -0.038 | 0.819 | 0.296 | 0.181 | -0.251 | 0.387 |
| CSHA | 0.025 | 0.885 | -0.025 | 0.877 | 0.199 | 0.375 | **-0.647** | **0.012** |
| NANO | -0.100 | 0.567 | **-0.332** | **0.032** | 0.263 | 0.238 | 0.353 | 0.215 |

Table S7 Correlation of pre-surgery evaluation score and perioperative nutritional status situation of patients finished the 3-month after surgery assessment. Nutritional status was measured by MNA in 1-month, 3-month 6-month and 1-year after surgery(p<0.05).
