# Supplementary material for: Comprehensive ability evaluation and trend analysis of patients with malignant intracranial tumors in the perisurgery period
Source: Brain Behav. 2021 Sep 23;11(11):e02192. doi: 10.1002/brb3.2192 (PMC8613416; doi:10.1002/brb3.2192)
Supplement: Supplementary file 8 — Table S8 [file BRB3-11-e02192-s008.docx]

| QLQ-BN20 Correlation analysis | | | | | | | | |
| --- | --- | --- | --- | --- | --- | --- | --- | --- |
|  | 1-month after surgery | | 3-month after surgery | | 6-month after surgery | | 1-year after surgery | |
|  | Correlation coefficient | Significance | Correlation coefficient | Significance | Correlation coefficient | Significance | Correlation coefficient | Significance |
| ADL | -0.163 | 0.334 | 0.311 | 0.139 | -0.028 | 0.865 | 0.200 | 0.494 |
| HAD-A | 0.281 | 0.092 | -0.021 | 0.921 | 0.238 | 0.140 | 0.367 | 0.196 |
| HAD-D | 0.198 | 0.241 | -0.130 | 0.546 | 0.255 | 0.112 | 0.261 | 0.368 |
| Frail | -0.124 | 0.463 | -0.181 | 0.397 | 0.093 | 0.567 | 0.043 | 0.885 |
| MNA | 0.110 | 0.517 | 0.299 | 0.156 | **-0.362** | **0.022** | -0.392 | 0.165 |
| MoCA | **0.479** | **0.003** | 0.359 | 0.085 | **-0.346** | **0.029** | 0-.068 | 0.817 |
| MMSE | **0.503** | **0.002** | 0.326 | 0.120 | -0.292 | 0.067 | 0.008 | 0.979 |
| CCI | 0.157 | 0.354 | 0.135 | 0.529 | -0.019 | 0.910 | -0.529 | 0.052 |
| CSHA | 0.010 | 0.955 | 0.124 | 0.563 | -0.021 | 0.899 | -0.036 | 0.904 |
| NANO | -0.194 | 0.251 | 0.316 | 0.133 | 0.167 | 0.302 | 0.022 | 0.942 |

Table S8 Correlation of pre-surgery evaluation score and perioperative prognosis situation of patients finished the 6-month after surgery assessment. Prognosis was measured by QLQ-BN20 in 1-month, 3-month, 6-month and 1-year after surgery(p<0.05).
