# Supplementary material for: Comprehensive ability evaluation and trend analysis of patients with malignant intracranial tumors in the perisurgery period
Source: Brain Behav. 2021 Sep 23;11(11):e02192. doi: 10.1002/brb3.2192 (PMC8613416; doi:10.1002/brb3.2192)
Supplement: Supplementary file 9 — Table S9 [file BRB3-11-e02192-s002.docx]

| QLQ-C30 Correlation analysis | | | | | | | | |
| --- | --- | --- | --- | --- | --- | --- | --- | --- |
|  | 1-month after surgery | | 3-month after surgery | | 6-month after surgery | | 1-year after surgery | |
|  | Correlation coefficient | Significance | Correlation coefficient | Significance | Correlation coefficient | Significance | Correlation coefficient | Significance |
| ADL | -0.070 | 0.682 | **0.419** | **0.046** | **0.380** | **0.014** | -0.059 | 0.834 |
| HAD-A | 0.223 | 0.185 | -0.392 | 0.064 | 0.280 | 0.081 | 0.246 | 0.377 |
| HAD-D | 0.165 | 0.330 | -0.303 | 0.160 | 0.288 | 0.071 | -0.224 | 0.423 |
| Frail | -0.147 | 0.386 | -0.189 | 0.389 | 0.298 | 0.058 | 0.156 | 0.579 |
| MNA | 0.142 | 0.403 | 0.279 | 0.197 | 0.045 | 0.780 | -0.244 | 0.381 |
| MoCA | **0.374** | **0.023** | 0.023 | 0.916 | -0.204 | 0.206 | -0.071 | 0.802 |
| MMSE | **0.513** | **0.001** | 0.080 | 0.717 | -0.301 | 0.059 | -0.016 | 0.956 |
| CCI | 0.275 | 0.100 | 0.041 | 0.851 | 0.257 | 0.124 | -0.187 | 0.505 |
| CSHA | -0.011 | 0.949 | -0.024 | 0.912 | 0.281 | 0.083 | -0.122 | 0.666 |
| NANO | -0.034 | 0.841 | 0.356 | 0.095 | 0.158 | 0.332 | -0.367 | 0.178 |

Table S9 Correlation of pre-surgery evaluation score and perioperative prognosis situation of patients finished the 6-month after surgery assessment. Prognosis was measured by QLQ-C30 in 1-month, 3-month, 6-month and 1-year after surgery(p<0.05).
