# Supplementary material for: Comprehensive ability evaluation and trend analysis of patients with malignant intracranial tumors in the perisurgery period
Source: Brain Behav. 2021 Sep 23;11(11):e02192. doi: 10.1002/brb3.2192 (PMC8613416; doi:10.1002/brb3.2192)
Supplement: Supplementary file 10 — Table S10 [file BRB3-11-e02192-s010.docx]

| MNA Correlation analysis | | | | | | | | |
| --- | --- | --- | --- | --- | --- | --- | --- | --- |
|  | 1-month after surgery | | 3-month after surgery | | 6-month after surgery | | 1-year after surgery | |
|  | Correlation coefficient | Significance | Correlation coefficient | Significance | Correlation coefficient | Significance | Correlation coefficient | Significance |
| QLQ BN20 | -0.105 | 0.534 | -0.015 | 0.946 | **-0.330** | **0.041** | 0.113 | 0.688 |
| QLQ C30 | -0.267 | 0.110 | -0.026 | 0.904 | -0.200 | 0.215 | 0.124 | 0.661 |
| ADL | -0.153 | 0.365 | -0.294 | 0.163 | -0.054 | 0.741 | 0.213 | 0.447 |
| HAD-A | -0.209 | 0.214 | 0.042 | 0.845 | **-0.322** | **0.043** | -0.043 | 0.878 |
| HAD-D | -0.145 | 0.392 | 0.017 | 0.937 | **-0.487** | **0.001** | 0.158 | 0.573 |
| Frail | -0.229 | 0.173 | 0.181 | 0.398 | -0.027 | 0.870 | -0.433 | 0.107 |
| MoCA | 0.141 | 0.405 | -0.358 | 0.086 | 0.049 | 0.765 | 0.315 | 0.253 |
| MMSE | 0.117 | 0.489 | **-0.442** | **0.030** | 0.159 | 0.328 | 0.059 | 0.835 |
| CCI | -0.157 | 0.353 | 0.192 | 0.369 | -0.063 | 0.712 | -0.182 | 0.517 |
| CSHA | -0.267 | 0.110 | 0.157 | 0.463 | **-0.466** | **0.003** | -0.131 | 0.643 |
| NANO | -0.070 | 0.683 | -0.068 | 0.752 | -0.160 | 0.325 | 0.273 | 0.325 |

Table S10 Correlation of pre-surgery evaluation score and perioperative nutritional status situation of patients finished the 6-month after surgery assessment. Nutritional status was measured by MNA in 1-month, 3-month 6-month and 1-year after surgery(p<0.05).
